# Upregulation of NETO2 expression correlates with tumor progression and poor prognosis in colorectal carcinoma

**DOI:** 10.1186/s12885-015-2018-y

**Published:** 2015-12-23

**Authors:** Liang Hu, Hai-Yang Chen, Jian Cai, Guang-Zhen Yang, Dan Feng, Yan-Xia Zhai, Hui Gong, Chen-Ye Qi, Yu Zhang, Hao Fu, Qing-Ping Cai, Chun-Fang Gao

**Affiliations:** 1 Anal-Colorectal Surgery Institute, 150th Hospital of PLA, Luoyang, China; 2 Department of Oncology, 150th Hospital of PLA, Luoyang, China; 3 Department of Colorectal Surgery, 150th Hospital of PLA, Luoyang, China; 4 Department of Clinical Laboratory, 150th Hospital of PLA, Luoyang, China; 5 Department of Oncology, Changhai Hospital, Second Military Medical University, Shanghai, China; 6 Department of Gastrointestine Surgery, Changzheng Hospital, Second Military Medical University, Shanghai, China

**Keywords:** NETO2, Colorectal carcinoma, Survival, Prognosis, Biomarker

## Abstract

**Background:**

Neuropilin and tolloid-like 2 (NETO2) has been found to be overexpressed in different human cancers, but its expression pattern and clinical relevance in colorectal carcinoma (CRC) remains unknown.

**Methods:**

Real-time quantitative PCR, western blot and immunohistochemistry analyses were used to analyze the expression of NETO2 in CRC clinical samples. The correlation of NETO2 expression with clinicopathologic features was estimated in a cohort containing 292 patients with primary CRC. Kaplan-Meier and Cox proportional hazards regression analyses were used to assess the prognostic value of NETO2 expression in CRC.

**Results:**

The expression of NETO2 was frequently upregulated in CRC clinical samples at both the mRNA and protein levels, and its upregulation was significantly correlated with poor tumor differentiation (*p* = 0.013), advanced local invasion (*p* = 0.049), increased lymph node metastasis (*p* = 0.009), advanced TNM stage (*p* = 0.041) and increased patient death (*p* = 0.001). Kaplan-Meier analysis of the complete study cohort revealed that patients with high-NETO2 tumors had a significantly shorter disease-specific survival (DSS) than those with low-NETO2 tumors (*p* < 0.001). Importantly, high levels of NETO2 protein predicted poor DSS for patients with early stage tumors (*p* = 0.027) and for those with advanced stage tumors (*p* = 0.020). Furthermore, multivariate analyses indicated that increased NETO2 expression was an independent unfavorable prognostic factor for patients with early stage tumors (hazard ratio [HR] = 1.937, 95 % CI = 1.107-3.390, *p* = 0.021) as well as patients with advanced stage tumors (HR = 2.241, 95 % CI = 1.245-4.035, *p* = 0.007).

**Conclusions:**

Our findings suggest that NETO2 upregulation could serve as a potential biomarker for the prediction of advanced tumor progression and unfavorable prognosis in patients with CRC.

## Background

Colorectal carcinoma (CRC) is the third most commonly diagnosed cancer in males and the second in females, with an estimated 1.4 million cases and 693,900 deaths occurring in 2012 [1]. Due to changes in dietary patterns and risk factors of lifestyle, the incidence of CRC has continued to increase in historically low-risk regions including several countries in Eastern Europe and China during the past decades [2–4]. The prognosis of CRC patients has shown only limited improvement despite advances in treatment approaches over the past few years, and the 5-year relative survival has remained less than 50 % in low-income countries [5, 6]. Currently, stage at diagnosis is still the most important prognostic indicator, and classification according to TNM stage provides valuable prognostic information and guides therapy decisions for CRC patients. However, clinical outcome of CRC patients after surgical resection varies greatly, even when patients are assigned to the same TNM category [7, 8]. Consequently, there is an urgent need to identify novel biomarkers to improve prognosis prediction for patients with CRC.

Neuropilin and tolloid-like 2 (NETO2), a single-pass transmembrane protein and, along with its only paralog NETO1, belongs to the unique subfamily of CUB domain- and LDLa-containing proteins [9]. Up to now, most studies have implicated the NETO2 gene in neuron-specific processes. It has been identified that NETO2 and NETO1 proteins function as auxiliary subunits of neuronal kainate receptors (KARs), which play important roles in excitatory synaptic transmission in the vertebrate brain [10], and modulate the kinetics of KARs by slowing desensitization or accelerating recovery from desensitization for these receptors [11, 12]. Therefore, it is proposed that NETO2, as well as NETO1, may provide an alternative target for the development of new drugs regulating KARs and brain function [13]. Mechanistic investigations have revealed that NETO2 could interact with the scaffolding protein Glutamate receptor-interacting protein (GRIP) and regulate synaptic abundance of KARs [14]. In addition, NETO2 has been demonstrated to be a K^+^–Cl^−^ cotransporter (KCC2) interacting protein and required for neuronal Cl^−^ regulation in hippocampal neurons [15]. Although NETO2 was initially believed to be a brain-specific protein [9, 11], a recent study conducted by Oparina et al. revealed that NETO2 mRNA expression was also detectable in a variety of normal non-neural tissues and upregulated in several types of cancers including renal, lung, colon and cervical carcinomas [16]. Moreover, they provided evidence that the NETO2 mRNA level could be a potential marker for early diagnosis in renal cancer and non-small cell lung cancer. These new findings encourage further investigation of its potential clinical significance in human malignancies.

Since the expression pattern and clinical relevance of NETO2 has not been investigated in human CRC, in the present study, we determined both the mRNA and protein expression levels of NETO2 in CRC clinical samples and further analyzed the correlation of NETO2 expression with clinicopathologic features and with patient survival based on tumor stage. Our results demonstrated that increased expression of NETO2 was correlated with tumor progression and might serve as an independent unfavorable prognostic indicator for patients with CRC.

## Methods

### Patients and specimens

Formalin-fixed paraffin-embedded tissue specimens from 292 stages I–III CRC patients who received curative surgery in our hospital (150th Hospital of the People's Liberation Army (PLA), Luoyang, China) from July 2006 to December 2009 were retrieved for immunohistochemistry. The study cohort consisted of CRC patients with typical adenocarcinoma histology as confirmed by pathological analysis. Distribution of the continuous variables of the study cohort was listed in Table 1. Detailed clinicopathologic characteristics of the patients were listed in Table 2. The follow-up period was defined as the interval from the date of surgery to the date of death or last follow-up. The latest follow-up was updated in September 2014, and the median follow-up time of the study cohort was 66 months (range, 1–98 months). Patients alive at the end of follow-up were censored. Disease-specific survival (DSS) was defined as the interval from the date of surgery to the date that patient died from CRC-related causes. Patients were excluded from the study cohort with the following exclusion criteria: previously received any anticancer therapy; impaired heart, lung, liver, or kidney function; previous malignant disease; died from diseases other than CRC or from unexpected events. TNM staging was classified according to the American Joint Committee on Cancer staging manual (seventh edition). 5 fluoruracil-based adjuvant chemotherapy was given to all stage III patients and a subgroup of stage II patients who had at least one of the following risk factors: pT4, bowel obstruction or perforation, poorly differentiated tumors, or less than 12 lymph nodes discovered after surgery.

**Table 1:** Distribution of continuous variables of the study cohort (*n* = 292)

Variable	Median	Mean ± SEM	Range	Percentile	
				25^th^	75^th^
Age (years)	66.0	65.5 ± 0.7	30.0-96.0	58.0	74.0
Tumor size (cm)	5.0	5.4 ± 0.2	1.5-15.0	4.0	6.0
Follow-up time (months)	66.0	56.3 ± 1.6	1.0-98.0	31.0	77.0
DSS time (months)	26.0	28.6 ± 1.7	1.0-80.0	13.0	42.0

*Abbreviations*: SEM, Standard error of the mean; DSS, Disease-specific survival

**Table 2:** Association between NETO2 expression and clinicopathologic characteristics of CRC patients in the study cohort

Characteristics		No. of patients (%)	NETO2 expression		
			Low (%)	High (%)	*P* value ^a^
		(*n* = 292)	(*n* = 115)	(*n* = 177)	
Age (years)					0.864
	<60	88 (30.1)	34 (29.6)	54 (30.5)	
	≥60	204 (69.9)	81 (70.4)	123 (69.5)	
Sex					0.860
	Female	111 (38.0)	43 (37.4)	68 (38.4)	
	Male	181 (62.0)	72 (62.6)	109 (61.6)	
Tumor location					0.227
	Right colon	88 (30.1)	31 (27.0)	37 (20.9)	
	Left colon	71 (24.4)	34 (29.5)	57 (32.2)	
	Rectum	133 (45.5)	50 (43.5)	83 (46.9)	
Differentiation grade					**0.013**
	Well	20 (6.8)	12 (10.4)	8 (4.5)	
	Moderate	222 (76.0)	91 (79.2)	131 (74.0)	
	Poor	50 (17.2)	12 (10.4)	38 (21.5)	
Tumor size (cm)					0.446
	<5	114 (39.0)	48 (41.7)	66 (37.3)	
	≥5	178 (61.0)	67 (58.3)	111 (62.7)	
Local invasion					**0.049**
	T_1_-T_2_	48 (16.4)	25 (21.7)	23 (13.0)	
	T_3_-T_4_	244 (83.6)	90 (78.3)	154 (87.0)	
Lymph node metastasis					**0.009**
	N_0_	164 (56.2)	74 (64.3)	90 (50.8)	
	N_1_	101 (34.6)	37 (32.2)	64 (36.2)	
	N_2_	27 (9.2)	4 (3.5)	23 (13.0)	
TNM stage					**0.041**
	I	40 (13.7)	21 (18.3)	19 (10.7)	
	II	124 (42.5)	53 (46.1)	71 (40.1)	
	III	128 (43.8)	41 (35.6)	87 (49.2)	
No. of examined lymph nodes					0.655
	<12	152 (52.1)	58 (50.4)	94 (53.1)	
	≥12	140 (47.9)	57 (49.6)	83 (46.9)	
Bowel obstruction/perforation					0.537
	No	282 (96.6)	112 (97.4)	170 (96.0)	
	Yes	10 (3.4)	3 (2.6)	7 (4.0)	
Adjuvant chemotherapy					0.060
	No	98 (33.6)	46 (40.0)	52 (29.4)	
	Yes	194 (66.4)	69 (60.0)	125 (70.6)	
Death					**0.001**
	No	168 (57.5)	80 (69.6)	88 (49.7)	
	Yes	124 (42.5)	35 (30.4)	89 (50.3)	

^**a**^Pearson chi-square test or Fisher exact test was used for comparison between subgroups. Bold type indicates statistical significance

A set of 57 paired fresh-frozen CRC samples obtained from stages I–III primary CRC patients who received curative surgery in our hospital from April 2013 to June 2013 were used for quantitative polymerase chain reaction (qPCR) analysis. An independent set of 24 paired fresh-frozen CRC samples obtained from stages I-III CRC patients who received curative surgery in our hospital from July 2013 to August 2013 were used for Western blot analysis. Written informed consent was obtained from each patient and this study was approved by the institutional Ethics Committee of our hospital.

### Real-time qPCR analysis

Real-time qPCR analysis was performed as described previously [17]. Briefly, total RNAs were isolated from frozen specimens using TRIzol Reagent (Ambion, 80706, USA). Reverse transcription was performed using RevertAidTM First Strand cDNA Synthesis Kit (Thermo Scientific, K1622, Lithuania) according to the manufacturer’s instructions. qPCR was performed on ABI Prism 7500 Sequence Detection System with SYBR Premix Ex Taq™ II (Takara, RR820A, Japan) using the 2^-ΔΔCT^ method. Gene expression results were normalized by internal control β-actin. The primers used in this study are as follows: NETO2 (NM_001201477.1) forward, 5'-AGCTGCTCCACGTCAAAGAA-3'; reverse, 5'- GCTCCCGAGAGCTCGAA-3'; β-actin forward, 5'-AATCGTGCGTGACATTAAGGAG-3'; reverse, 5’-ACTGTGTTGGCGTACAGGTCTT-3'. Each sample was tested in triplicate.

### Western blot analysis

Western blotting was performed as described previously [18]. Briefly, tumor specimens were prepared in lysis buffer [Tris–HCl (20 mM), pH 7.4, NaCl (150 mM), glycerol (10 %), Nonidet P-40 (0.2 %), EDTA (1 mM), EGTA (1 mM), PMSF (1 mM), NaF (10 mM), aprotinin (5 mg/ml), leupeptin (20 mM), and sodium orthovanadate (1 mM)] and centrifuged at 12,000 g for 30 min. Protein concentrations were measured using the BCA assay. Immunoblotting was performed using a primary antibody specific for NETO2 (Abcam, ab109288) and immunocomplexes were incubated with goat anti-rabbit fluorescein-conjugated secondary antibodies, and then detected using an Odyssey fluorescence scanner (Li-Cor, Gene Company). β-actin was used as a loading control (Santa Cruz Biotechnology, sc-47778).

### Immunohistochemistry and immunocytochemistry

Immunohistochemistry of paraffin-embedded tissue sections was performed as described previously [19]. Briefly, sections were deparaffinized and rehydrated. The endogenous peroxidase activity was blocked with 3 % H_2_O_2_ for 10 min. Antigens were retrieved with citrate buffer (10 mM, pH 6.0) for 15 min at 100 °C in a microwave oven. After blocking, the sections were incubated with a primary anti-NETO2 antibody (Abcam, ab171651) with 1:200 dilution at 4 °C overnight in a moist chamber followed by incubated with an anti-rabbit peroxidase-conjugated secondary antibody (Santa Cruz) at room temperature for 30 min. Finally, the visualization signal was developed with diaminobenzidine (Dako) and the slides were counterstained with hematoxylin. Immunocytochemistry was performed using human CRC cell line HCT15 as a positive control for immunostaining of NETO2 and HCT116 as a negative control.

Stained sections were evaluated in a blinded manner without prior knowledge of the clinical data using the German immunoreactive score (IRS) as described previously [20]. Briefly, staining intensity was graded as “0” (negative), “1” (weak), “2” (moderate) and “3” (strong); staining extent was graded as “0” (<5 %), “1” (5–25 %), “2” (25–50 %), “3” (50–75 %) or “4” (>75 %). Values of the staining intensity and the staining extent were multiplied as a final IRS of NETO2 expression. Using this method of assessment, we evaluated NETO2 expression in CRC tissues by the IRS of 0, 1, 2, 3, 4, 6, 8, 9, or 12. The median value of the IRS was chosen as the cut-off for high and low NETO2 expression levels based on a measure of heterogeneity according to the log-rank test with respect to DSS, as described previously [21]. An IRS of ≥ 6 was used to indicate tumors with high NETO2 expression and an IRS of < 6 was used to define tumors with low NETO2 expression. Discrepancies in the IRS were resolved by discussing together with other pathologists to reach a consensus.

### Statistical analysis

Pearson chi-square test or Fisher exact test was used to analyze the relationship between NETO2 expression and clinical features. Kaplan-Meier analysis with log-rank test was used to compare patients’ survival between subgroups. The effect of each variable on survival was determined by the Cox multivariate regression analysis. All statistical analyses were carried out using SPSS PASW Statistics 18.0 software (SPSS, Inc., Chicago, IL), and *p* value < 0.05 was considered to be statistically significant.

## Results

### Overexpression of NETO2 in primary CRC tissues

**Fig. 1:**
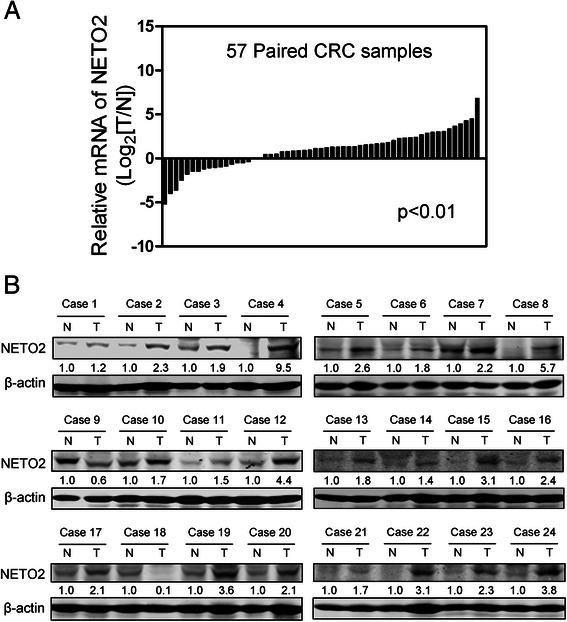
Expression of NETO2 in primary CRC tissues. **a** NETO2 mRNA expression in 57 paired human CRC and corresponding adjacent normal mucosa specimens were determined by real-time qPCR method. Gene expression results were normalized by internal control β-actin. (T, tumor tissues; N, adjacent normal tissues). **b** Protein expression levels of NETO2 in an independent set of 24 paired CRC and corresponding adjacent normal specimens were determined by Western blot analysis. β-actin was used as a loading control. The relative protein expression of NETO2 was quantified and normalized to β-actin. Each N was arbitrarily designated 1.0. (T: Tumor tissues; N: adjacent normal tissues, T vs N, *p* < 0.001)

The expression levels of NETO2 mRNA in 57 paired CRC and corresponding adjacent normal mucosa specimens were quantified by real-time qPCR method. The results showed that NETO2 mRNA expression was significantly upregulated in the cancerous tissues compared with adjacent normal counterparts, in which 52.6 % (30/57) of the cancerous specimens tested showed a significant increase (over 2-fold) in NETO2 mRNA level (Fig. 1a, *p* < 0.01). To determine the protein levels of NETO2 in CRC, an independent set of 24 paired CRC and corresponding adjacent normal mucosa specimens were subjected to Western blot assay. As shown in Fig. 1b, the protein levels of NETO2 were also significantly higher in cancerous tissues than in adjacent normal counterparts (*p* < 0.001).

**Fig. 2:**
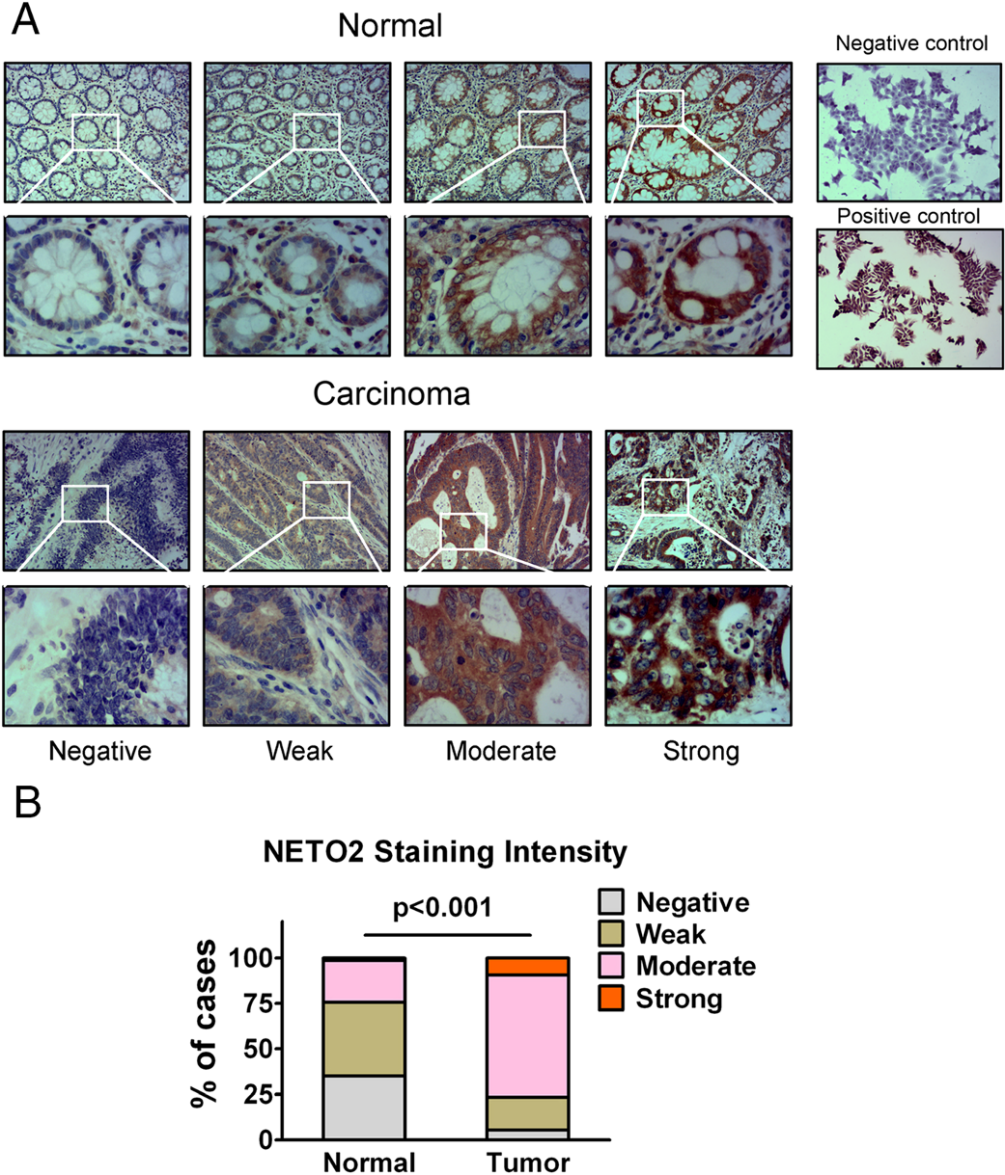
Immunostaining of NETO2 protein in human CRC and adjacent normal tissues. **a** Representative immunohistochemical expression patterns of NETO2 in cancerous and adjacent normal mucosa specimens were shown. (Magnification, upper panel, ×100; lower panel, ×400) Right panel: human CRC cell line HCT116 cells were used as a negative control and HCT15 cells were used as a positive control for immunostaining of NETO2 protein. (Magnification × 100) (**b**) Percentage of cases with different staining intensity of NETO2 in the tumor or adjacent normal tissues in the study cohort. (*p* < 0.001)

To further evaluate the phenotypic expression of NETO2 protein in CRC clinical samples, immunohistochemical analysis was performed in another independent set of 292 pairs of CRC specimens and adjacent normal tissues. The immunoreactivity of NETO2 protein was observed primarily in the cytoplasm. The staining intensities were classified into four levels: level 1 with negative staining, level 2 with weak staining, level 3 with moderate staining, and level 4 with strong staining (Fig. 2a). Overall, 9.2 % (27/292) of the cancerous specimens showed strong staining, 67.5 % (197/292) of the cases showed moderate staining, 18.2 % (53/292) of the cases showed weak staining, and only 5.1 % (15/292) of the cases showed negative staining of NETO2 protein. In striking contrast, 34.9 % (102/292) of the adjacent normal mucosa tissues showed negative staining, 40.8 % (119/292) of the cases showed weak staining, 22.9 % (67/292) of cases showed moderate staining, and only 1.4 % (4/292) of cases showed strong staining of NETO2 (Fig. 2b, *p* < 0.001). Thus, NETO2 was frequently overexpressed in primary CRC tissues.

### Correlation of NETO2 expression with clinicopathologic features

To evaluate the association between NETO2 expression levels and clinicopathologic characteristics, the 292 patients were classified into high and low NETO2 expression subgroups with the median IRS value as the cut-off. As shown in Table 2, high expression of NETO2 protein was significantly correlated with poor tumor differentiation (*p* = 0.013), advanced local invasion (*p* = 0.049), increased lymph node metastasis (*p* = 0.009), advanced TNM stage of the disease (*p* = 0.041), and increased death rate of patients (*p* = 0.001). While, there were no significant associations between NEOT2 expression and patient age (*p* = 0.864), sex (*p* = 0.860), tumor location (*p* = 0.227), tumor size (*p* = 0.446), number of examined lymph nodes (*p* = 0.655), preoperative bowel obstruction or perforation (*p* = 0.537), or adjuvant chemotherapy (*p* = 0.060).

### Prognostic values of NETO2 expression for patients with CRC

**Fig. 3:**
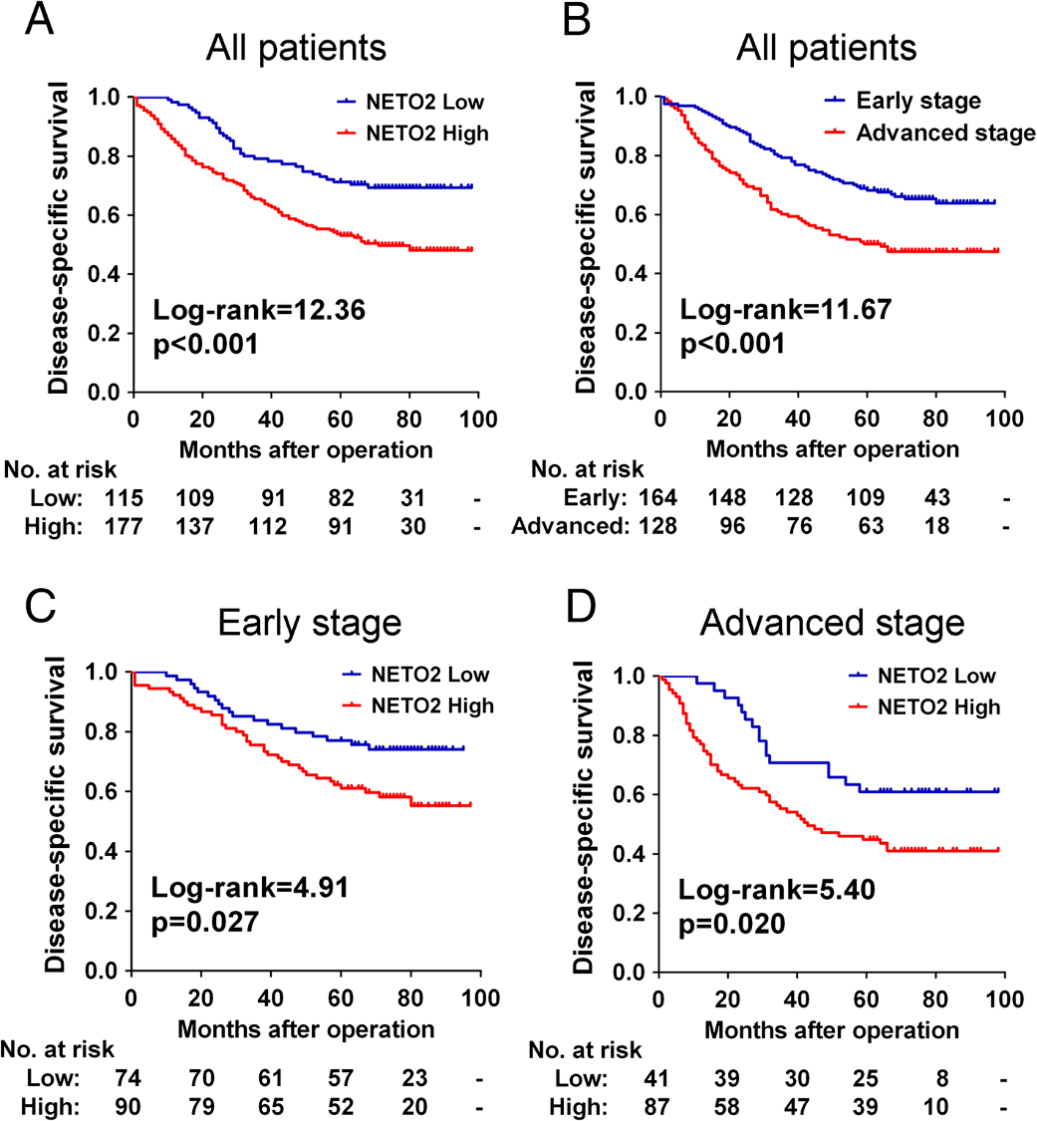
Kaplan-Meier survival analysis for CRC patients. **a** Kaplan-Meier curves for disease-specific survival of all CRC patients in the study cohort according to NEOT2 expression status. **b** Kaplan-Meier curves for disease-specific survival of all CRC patients according to TNM stage. **c**-**d** Kaplan-Meier curves for disease-specific survival of patients with early stage tumors (**c**) or advanced stage tumors (**d**) according to NETO2 expression status. The p-value was determined using the log-rank test. The absolute number of patients at risk in each subgroup is listed below

Of the 292 patients, 124 had died from disease progression within the follow-up period. The cumulative 5-year disease-specific survival (DSS) rate was 60.3 %. Kaplan-Meier analysis of the complete study cohort revealed that patients with high-NETO2 tumors had a significantly shorter DSS than those with low-NETO2 tumors (Fig. 3a, *p* < 0.001). The cumulative 5-year DSS rate was 71.3 % in patients with low-NETO2 tumors, whereas it was only 53.1 % in those with high-NETO2 tumors. In our cohort, patients who had advanced stage (stage III) tumors had a significantly unfavorable prognosis compared with those who had early stage (stages I-II) tumors (Fig. 3b, *p* < 0.001).

Survival analyses based on tumor stage (early and advanced) demonstrated that high expression of NETO2 significantly predicted poor DSS in patients with early stage tumors (Fig. 3c, *p* = 0.027). Similarly, high levels of NETO2 also significantly predicted poor DSS in patients with advanced stage tumors (Fig. 3d, *p* = 0.020).

To assess whether NETO2 expression represents an independent prognostic indicator in CRC, the effect of each variable on survival was determined by the Cox regression analysis. Univariate analyses revealed that NETO2 expression (HR = 1.986, 95 % CI = 1.343-2.937, *p* = 0.001), TNM stage (HR = 1.832, 95 % CI = 1.286-2.609, *p* = 0.001), tumor differentiation grade (HR = 1.691, 95 % CI = 1.103-2.591, *p* = 0.016), and patient age (HR = 1.674, 95 % CI = 1.098-2.551, *p* = 0.017) were significantly associated with DSS (Table 3). The variables that significantly correlated with survival in the univariate analysis were further assessed by multivariate analysis. The results of the multivariate analysis confirmed that NETO2 expression (HR = 1.924, 95 % CI = 1.300-2.848, *p* = 0.001), TNM stage (HR = 1.878, 95 % CI = 1.313-2.685, *p* = 0.001), and patient age (HR = 1.875, 95 % CI = 1.225- 2.871, *p* = 0.004) were independent prognostic factors for patients with CRC (Table 3).

**Table 3:** Univariate and multivariate analyses of prognostic factors for disease-specific survival of CRC patients in the study cohort

Variables	Categories	Univariate analysis			Multivariate analysis		
		HR	95 % CI	*P* value^a^	HR	95 % CI	*P* value^a^
Age (years)	≥60/<60	1.674	1.098-2.551	**0.017**	1.875	1.225-2.871	**0.004**
Sex	Male/female	1.141	0.791-1.646	0.480			
Tumor location	Colon/rectum	0.960	0.674-1.367	0.820			
Tumor size (cm)	≥5/<5	1.161	0.807-1.672	0.421			
No. of examined lymph nodes	≥12/< 12	0.952	0.669-1.354	0.783			
Bowel obstruction/perforation	Yes/no	0.962	0.355-2.607	0.940			
Adjuvant chemotherapy	Yes/no	1.246	0.851-1.825	0.259			
Differentiation grade	Poor well + moderate	1.691	1.103-2.591	**0.016**			
TNM stage	III/I+ II	1.832	1.286-2.609	**0.001**	1.878	1.313-2.685	**0.001**
NETO2 expression	High/low	1.986	1.343-2.937	**0.001**	1.924	1.300-2.848	**0.001**

*Abbreviations*: HR, hazard ratio; 95 % CI, 95 % confidence interval

^a^Bold type indicates statistical significance

The independent prognostic significance of NETO2 expression on CRC-specific survival based on tumor stage was further evaluated with a Cox regression model. The results showed that increased NETO2 expression was an independent indicator of a poor prognosis for patients with early stage tumors (HR = 1.937, 95 % CI = 1.107-3.390, *p* = 0.021) as well as for those with advanced stage tumors (HR = 2.241, 95 % CI = 1.245-4.035, *p* = 0.007). For patients with advanced stage tumors, age (HR = 2.229; 95 % CI = 1.264-3.932, *p* = 0.006) and number of examined lymph nodes (HR = 0.584; 95 % CI = 0.351-0.973, *p* = 0.039) were also significantly associated with patient survival (Table 4).

**Table 4:** Multivariate analyses of prognostic factors for disease-specific survival of patients with early or advanced stage tumors in the study cohort

Variables	Categories	Early stage			Advanced stage		
		HR	95 % CI	*P* value^b^	HR	95 % CI	*P* value^b^
Age (years)	≥60/<60	1.705	0.860-3.381	0.127	2.229	1.264-3.932	**0.006**
Sex	Male/female	0.818	0.462-1.447	0.489	1.575	0.934-2.658	0.089
Tumor location	Colon/rectum	1.095	0.802-1.495	0.569	0.968	0.727-1.290	0.826
Tumor size (cm)	≥5/<5	0.920	0.536-1.579	0.762	1.558	0.915-2.652	0.103
No. of examined lymph nodes	≥12/< 12	1.525	0.785-2.964	0.213	0.584	0.351-0.973	**0.039**
Bowel obstruction/perforation	Yes/no	1.365	0.175-10.672	0.767	0.689	0.209-2.269	0.541
Adjuvant chemotherapy^a^	Yes/no	0.808	0.397-1.644	0.556			
Differentiation grade	Poor/well + moderate	0.690	0.207-2.298	0.545	1.591	0.946-2.678	0.080
NETO2 expression	High/low	1.937	1.107-3.390	**0.021**	2.241	1.245-4.035	**0.007**

*Abbreviations*: HR, hazard ratio; 95 % CI, 95 % confidence interval

^a^As all advanced stage patients had received adjuvant chemotherapy, adjuvant chemotherapy was not enrolled into the multivariate analysis

^b^Bold type indicates statistical significance

## Discussion

NETO2 was initially reported to be specifically expressed in the nervous system and previous investigations of this gene focused almost entirely on the neurobiological aspects, but subsequent studies revealed that it is dysregulated in several pathological conditions. Using a genome-wide transcriptional profiling analysis, Calicchio et al. reported that NETO2 belongs to the genes overexpressed in proliferating hemangiomas relative to normal placental vessels [22]. In addition, Horak et al. found that it could be downregulated by the metastasis suppressor gene Nm23-H1 in breast carcinoma cell lines [23]. Notably, Oparina et al. recently demonstrated that the NETO2 mRNA is frequently overexpressed in a number of human neoplasms and might be a tool to support the early diagnosis of renal and lung carcinomas [16]. These findings indicate that NETO2 may have potential significance in cancer pathobiology. Nevertheless, the clinical relavance of NETO2 expression has not been assessed in CRC.

The present study, to our knowledge, is the first to report the phenotypic expression pattern of NETO2 and its clinical significance in CRC. Using real-time qPCR and Western blot analysis, we found that NETO2 was frequently overexpressed in primary CRC samples at both the mRNA and protein levels. Our results are consistent with the previous findings of Oparina et al. at the mRNA level [16]. Of note, we observed a significant increase in NETO2 mRNA level (over 2-fold) in 52.6 % (30/57) of the cancerous specimens tested, whereas Oparina et al. reported that only 40 % (4/10) of colon cancer samples showed substantially increased expression of NETO2 mRNA. This discrepancy may result from the difference in the sample size, ethnic group or endogenous control gene used for normalization. Notably, as these tumor tissues were not isolated by laser capture microdissection technology, the inclusion of non-epithelial cells not expressing NETO2 in PCR and Western blot experiments may interfere with the evaluation of NETO2 expression in CRC and adjacent normal tissues. Nevertheless, our subsequent immunohistochemical analysis of 292 paired CRC specimens demonstrated that expression of NETO2 protein was significantly upregulated in the cancerous colorectal epithelial cells compared with the adjacent normal counterparts, in which 76.7 % (224/292) of the cancerous tissues presented moderate-strong staining of NETO2 protein, whereas only 24.3 % (71/292) of the adjacent normal tissues showed virtually the same immunoreactivity. Thus, our results unambiguously confirmed the significant upregulation of NETO2 expression in CRC. In addition, unlike the reported transmembrane localization in neurons [9, 11], we found that NETO2 protein was mainly localized in the cytoplasm. This observation is in agreement with the results of the Protein Atlas large-scale immunohistochemical study of human proteins, which displayed a cytoplasmic staining of NETO2 in human normal colorectal tissues [http://www.proteinatlas.org/ENSG00000171208]. Hence, although *NETO2* gene was initially found only expressed in brain of human and mice by Northern blotting and in situ hybridization analysis [9], the data collected from Oparina et al. and our group demonstrated that it is also expressed in non-neural normal and neoplastic tissues. Moreover, the observed significant upregulation of NETO2 expression in malignant diseases is in accordance with similar cases where expression of certain neuron-specific proteins is activated in oncogenesis [24, 25]. However, the molecular basis and biological relevance of NETO2 overexpression in CRC is currently unclear and needs further investigation.

Interestingly, according to our results, increased expression of NETO2 protein in CRC was significantly correlated with poor differentiation, advanced local invasion, increased lymph node metastasis and advanced TNM stage, indicating that NETO2 may be involved in the progression of CRC. It should be noted that, although our data demonstrated an association of NETO2 expression with aggressive clinical phenotypes, whether NETO2 plays a functional role in the progression of CRC needs to be carefully determined. Since the increase in NETO2 expression could also be the result of other factors that lead to cancer progression, rather than NETO2 overexpression being a contributing factor in cancer progression. Further in vitro and in vivo functional studies are warranted to address this issue.

The most important finding of the present study was the prognostic value of NETO2 in CRC patients. We observed a significant association between increased NETO2 protein expression and poor survival of CRC patients in both univariate and multivariate survival analyses. In addition, our results also demonstrated that TNM stage is an important prognostic factor in CRC, which is consistent with the well established adverse prognostic effect of tumor stage [26] and confirms that our cohort was representative and that the survival analyses were valid. Moreover, stage-based survival analyses revealed that increased expression of NETO2 protein in tumors not only significantly predicted poor DSS but also was an independent unfavorable prognostic indicator for patients with early stage tumors as well as for those with advanced stage tumors. These findings should be of particular interest especially for patients who have early stage tumors. It is well known that TNM staging has a great influence on CRC prognosis and is clinically accepted as a solid basis for therapeutic management. However, dilemmas are often raised with respect to the treatment of patients with early stage disease. Generally speaking, patients who had early stage CRCs have a favorable prognosis compared with those who had advanced stage CRCs. Nevertheless, a subgroup of patients with early stage disease have an increased risk of early recurrence and death [27]. Hence, it is of particular importance to identify this high-risk subgroup of patients for appropriate treatment. Thus, results from the present work suggest that NETO2 expression status could serve as a promising biomarker to classify patients with early stage tumors into distinct risk subgroups and guide individualized therapeutic strategy.

The present study had several limitations. Although our results revealed the clinicopathologic correlation and prognostic value of NETO2 protein expression in a cohort of CRC patients, the potential role of NETO2 in the development of CRC has not been elucidated. In addition, due to the limitation of follow-up period, the median survival time of patients with low-NETO2 tumors could not be obtained, thus, our current results could not accurately reflect the survival of patients in this subgroup. Besides, since the limited quantity of CRC tissue samples, three independent sets of CRC specimens were used with each set studied with each of the method (qPCR, Western, IHC). Therefore, the fact that different sets were employed makes impossible any direct comparison to specifically answer the question of whether there is a correlation between NETO2 expression in the mRNA and protein levels. Further studies are necessary to confirm our findings and clarify the function and mechanism of NETO2 in the development of CRC.

## Conclusions

We here provide evidence, for the first time, that NETO2 expression was frequently upregulated in CRC tissues at both the mRNA and protein levels. In addition, increased expression *o*f NETO2 was significantly associated with disease progression and poor postoperative outcome of CRC patients. Our results suggest that NETO2 might serve as a novel prognostic molecular marker for patients with CRC and encourage further investigation of its potential role in CRC pathobiology.

## Abbreviations

CRC: colorectal carcinoma
NETO2: neuropilin and tolloid-like 2
DSS: disease-specific survival
IHC: immunohistochemistry
IRS: immunoreactive score
KARs: kainate receptors
KCC2: K^+^–Cl^−^ cotransporter 2
GRIP: glutamate receptor-interacting protein
CI: confidence interval
HR: hazard ratio
